# Medical student knowledge regarding radiology before and after a radiological anatomy module: implications for vertical integration and self-directed learning

**DOI:** 10.1007/s13244-014-0346-0

**Published:** 2014-08-10

**Authors:** Kevin P. Murphy, Lee Crush, Eoin O’Malley, Fergus E. Daly, Colm M. P. O’Tuathaigh, Owen J. O’Connor, John F. Cryan, Michael M. Maher

**Affiliations:** 1 Department of Anatomy and Neuroscience, University College Cork, Cork, Ireland; 2 Department of Radiology, University College Cork, Cork, Ireland; 3 Department of Medicine, University College Cork, Cork, Ireland

**Keywords:** Radiology, Medical student, Radiologist, Radiographer, Radiation dose

## Abstract

**Objectives:**

To examine the impact that anatomy-focused radiology teaching has on non-examined knowledge regarding radiation safety and radiology as a specialty.

**Methods:**

First-year undergraduate medical students completed surveys prior to and after undertaking the first-year anatomy programme that incorporates radiological anatomy. Students were asked opinions on preferred learning methodology and tested on understanding of radiology as a specialty and radiation safety.

**Results:**

Pre-module and post-module response rates were 93 % (157/168) and 85 % (136/160), respectively. Pre-module and post-module, self-directed learning (SDL) ranked eighth (of 11) for preferred gross-anatomy teaching formats. Correct responses regarding radiologist/radiographer roles varied from 28-94 % on 16 questions with 4/16 significantly improving post-module. Identification of modalities that utilise radiation significantly improved for five of eight modalities post-module but knowledge regarding relative amount of modality-specific radiation use was variable pre-module and post-module.

**Conclusions:**

SDL is not favoured as an anatomy teaching method. Exposure of students to a radiological anatomy module delivered by senior clinical radiologists improved basic knowledge regarding ionising radiation use, but there was no improvement in knowledge regarding radiation exposure relative per modality. A possible explanation is that students recall knowledge imparted in didactic lectures but do little reading around the subject when the content is not examined.

**Teaching Points:**

• *Self-directed learning is not favoured as a gross anatomy teaching format amongst medical students.*

• *An imaging anatomy-focused module improved basic knowledge regarding ionising radiation use.*

• *Detailed knowledge of modality-specific radiation exposure remained suboptimal post-module.*

• *Knowledge of roles within a clinical radiology department showed little change post-module.*

## Introduction

Vertical integration and system-based learning are now central components of the curricula delivered to medical students [1–10]. Reported benefits of these methods include improved student learning, increased student satisfaction and interaction, enhanced applied knowledge and ultimately, greater preparedness for post-graduate employment. Anatomy remains a core topic for 1st-year medical students in this new environment, but the volume and method of delivery has changed such that problem-based and self-directed learning (SDL) are utilised to a greater degree than previously [1, 11–14]. In addition to this, imaging anatomy is increasingly acknowledged as important and the rapid advancements in cross-sectional imaging, including developments in computed tomography (CT) and magnetic resonance (MR) angiography, venography and cholangiography, now means that these images represent powerful tools for teaching human anatomy. As a result, at many medical schools there are moves to develop modules in imaging anatomy and for clinical radiologists to deliver modules in small and large group sessions. These developments are particularly seen in postgraduate/graduate entry medical programmes [15–20].

SDL is an important tool for life-long learning, which is an integral part of professional life as a medical doctor; hence SDL techniques are increasingly promoted at an early stage in Medical School. Due to differences in learning experience and methodology, different approaches may be required for undergraduate and postgraduate/graduate entry students. Undergraduate students in many jurisdictions are coming from an environment of traditional directly taught content that has a stronger emphasis on rote learning rather than SDL or critical thinking. On the other hand, postgraduate students already have experienced university education and are better equipped for SDL [21–23]. A number of studies have reported predominantly positive results on the impact of SDL promotion on students’ readiness and enthusiasm for same [24–31]. The level of non-examined, non-compulsory student knowledge as a marker of SDL success has not been explored.

For this purpose, we designed a student survey to test student awareness of extra-curricular non-taught knowledge of radiological anatomy and other elements of clinical radiology. The 1st-year curriculum in anatomy includes a 10-h module in imaging anatomy delivered by senior clinical radiologists. The imaging anatomy teaching incorporates didactic lectures in a large lecture theatre setting and small group interactive sessions in a modern anatomy laboratory. These sessions include a brief amount of background information regarding radiology in the form of a dedicated introductory lecture and references to diagnostic imaging procedures in all other sessions, but the core information delivery is with regard to radiological anatomy and modality recognition. This imaging anatomy module represents the first exposure of medical students to radiology. Instruction in radiology is integrated into all 5 years of the medical undergraduate programme and approximately 50 h of teaching is spread over each of the 5 years of medical school. SDL is also heavily promoted both in the anatomy syllabus and other modules. In the anatomy and radiological anatomy module, SDL resources are promoted via book lists, reference books, and departmental and online e-learning resources. We invited students to participate in an assessment conducted before commencing and after completing their first year of anatomy teaching in order to ascertain their knowledge with regard to (1) opinions on SDL in terms of teaching format preference, (2) radiation safety awareness and (3) comprehension of radiology as a specialty.

## Materials and methods

### Study design

The study was approved by the institutional ethics research committee. A descriptive quantitative cross-sectional study design was used. First-year direct entry medical students at our university were invited to participate. The first questionnaire was completed on admission to medical school during an introductory course delivered in the first week and prior to commencing the 1st-year course in anatomy. All students were informed of the purpose and ethical approval status of the study. The assessment was repeated 1 year later at the beginning of the 2nd medical year in a similar manner. The post-module questionnaire is presented in appendices 1–4. Questionnaires were distributed by hand to students at the beginning of each assessment and collected at the end of the session. Students self-administered the majority of the questions.

### Questionnaire design

The survey was designed by a multidisciplinary team of anatomists, clinical radiologists and medical educators. A large survey was designed which extensively evaluated perceptions of radiology in anatomy teaching. A section of this larger survey, which focused on radiation exposure and radiation protection associated with diagnostic imaging and interventional radiology, forms the basis of the current study. This component of the survey sought to evaluate extra-curricular non-taught elements of radiological anatomy curriculum. Data collected pertained to student demographics, preferred anatomy learning methods, opinions regarding radiology as a specialty and understanding of imaging modalities. A closed format was used, utilising mainly multiple-choice questions, for ease of analysis. Rank-style questions, Likert-style statements and binomial “yes/no” questions were utilised.

### Analysis

Data were coded and converted into appropriate variables and entered manually into an Excel spreadsheet. Data were then exported to the Statistical Package for Social Scientists (SPSS) version 20 (IBM, Armonk, NY). Descriptive and inferential statistics were employed. Chi-squared, Spearman’s correlation and Kendall’s correlation were used to analyse the data and detect associations, where appropriate.

## Results

### Demographics of respondents and opinions on SDL

Pre- and post-module response rates were 93 % (157/168) and 85 % (136/160), respectively. Almost two-thirds (63 %) of participants were female and the median age of respondents at the time of the second survey was 20 years (range, 18–38 years). Almost half (48.4 %) of participants were citizens of the country in which medical the school was located, with 44.6 % from an Asian country and the remainder (7 %) from one of five other countries. Four students in this direct entry programme had previous degrees; hence these students were omitted from analysis in order to ensure that experience with SDL that may have been learned from a previous degree programme did not influence results.

SDL was ranked as eighth (median) (out of 11) in terms of preferred teaching formats for gross anatomy both pre-module and post-module.

### Understanding of a clinical radiology department

**Fig. 1:**
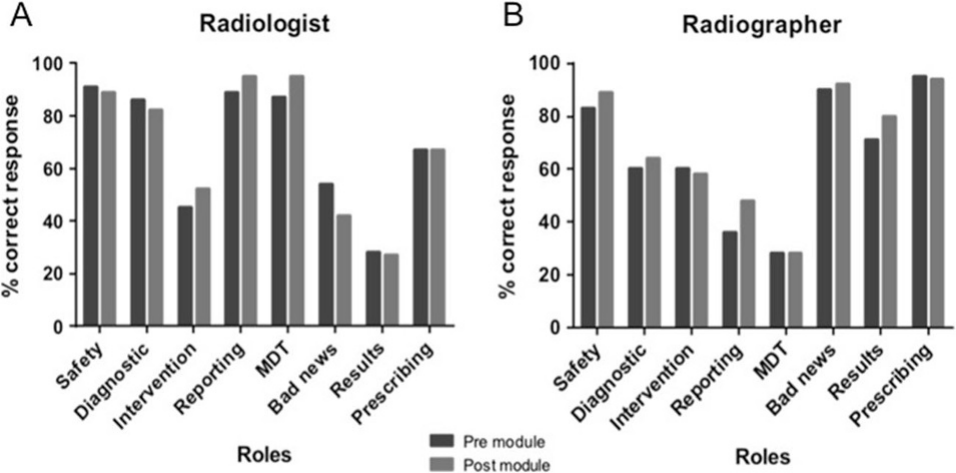
Student knowledge regarding the role of clinical radiologists (**a**) and radiographers (**b**). *Safety* patient safety and care, *Diagnostic* performing diagnostic studies, *Intervention* performing interventional procedures, *Reporting* reporting on diagnostic studies, *MDT* multidisciplinary team meeting attendance, *Bad news* breaking bad news to patients, *Results* giving results to patients after imaging studies, *Prescribing* prescribing medications regularly

Students were asked as to the role of radiologists and radiographers within a hospital and clinical radiology department (Fig. 1). Post-module, students correctly identified that radiologists interpret and report (94 % correct) imaging studies and work as part of a multidisciplinary team (94 % correct), but only 52 % were aware that radiologists performed interventional procedures and 67 % were aware that radiologists do not regularly prescribe medications. Less than 50 % of students knew that radiographers rarely attend multidisciplinary meetings or that they do not report studies (in the jurisdiction of the medical school). On follow-up, only 63 % were aware that radiographers perform diagnostic imaging studies such as plain radiography, CT and MR imaging. Ninety-one percent of students on the second questionnaire were aware that radiographers did not disclose bad news or imaging results to patients and that they do not prescribe medications. There were significant improvements between pre-module and post-module regarding knowledge of radiologist multidisciplinary team participation (*p* = 0.021), radiologist reporting (*p* = 0.006), radiographer non-reporting awareness (*p* = 0.027) and radiographer patient result delivery knowledge (*p* = 0.047). A significant deterioration in knowledge was seen with regard to the knowledge of infrequence of bad news delivery to patients by radiologists (*p* = 0.027).

### Radiation safety awareness

**Fig. 2:**
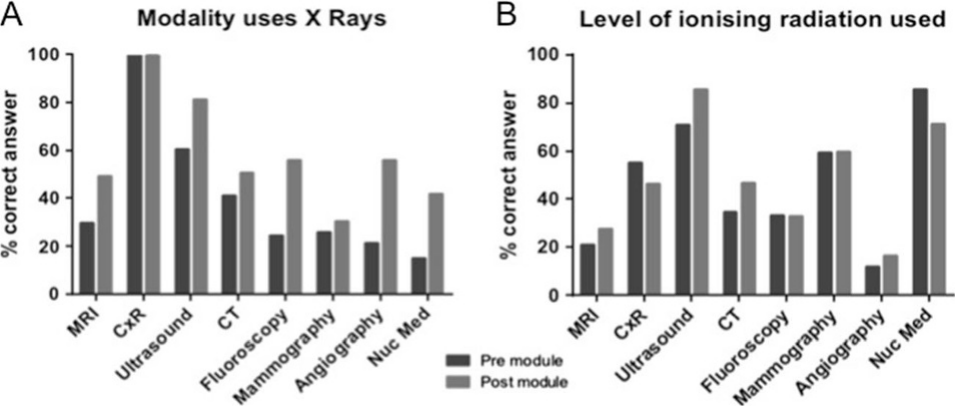
Student knowledge regarding modalities and imaging studies that involve exposure to ionising radiation (**a**) and the level of radiation used in these (**b**). *MRI* magnetic resonance imaging, *CxR* chest X-ray/radiograph, *Nuc Med* nuclear medicine imaging

Knowledge regarding radiation use in different imaging modalities and studies was assessed (Fig. 2). Almost all students were aware that a chest radiograph was associated with exposure to ionising radiation and 51 % post-module identified that CT utilised same. Post-module, 81 % and 50 % correctly identified that ultrasound and MR imaging respectively did not result in exposure to ionising radiation. Following module completion, greater numbers of students understood that fluoroscopy, angiography and nuclear medicine were associated with exposure to ionising radiation, though the percent of correct answers did not exceed 60 % for these entities. Knowledge regarding modalities that are associated with exposure to ionising radiation significantly improved on the second survey for all modalities (*p* < 0.05) apart from chest radiography, mammography (*p* = 0.551) and CT (*p* = 0.185).

With regard to the relative radiation exposures associated with individual imaging investigations, students were asked to rank the amount of radiation exposure per modality as “0” (no radiation), “1” (low level of radiation) or “2” (high level of radiation). In this question, 87 and 28 % of students correctly identified that ultrasound and MR imaging did not result in radiation exposure on follow-up. For the remaining imaging investigations, the level of awareness regarding relative radiation exposures was poorest for angiography (18 %) and best for nuclear medicine (71 %) on follow-up. Understanding regarding relative radiation exposure levels significantly disimproved for nuclear medicine (*p* = 0.003) and chest radiography (*p* = 0.044), whereas knowledge on relative radiation exposure significantly improved for CT (*p* = 0.016) and ultrasound (*p* < 0.001).

### Clinical radiology as a specialty

**Fig. 3:**
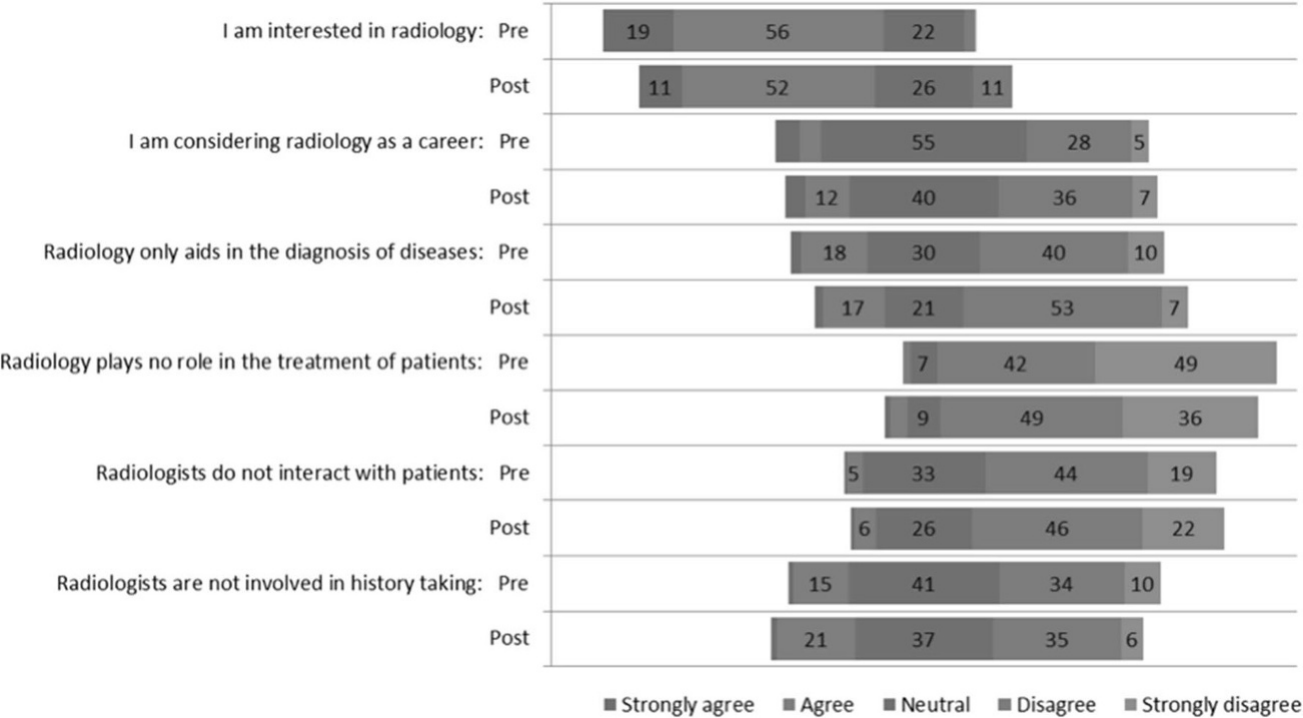
Pre-module and post-module opinions on clinical radiology statements with Likert responses

Pre-module and post-module opinions on clinical radiology statements with Likert responses are shown in Fig. 3. When students were asked opinions on the statement, “I am interested in radiology", pre-module 19 % , 56 % and 22 % of students strongly agreed, agreed or were neutral. Post-module, these figures changed to 11 % , 52 % and 26 % respectively in keeping with a significant decrease in interest (*p* = 0.003). In response to the statement, “I am considering radiology as a career”, 12.2 % agreed or strongly agreed pre-module, whereas 17.3 % did so post-module. This change did not reach significance.

## Discussion

Student’s knowledge regarding radiology as a clinical specialty and regarding awareness of radiation exposure associated with imaging investigations when pre-module and post-module surveys were examined. Students perceived clinical radiology as an important specialty and the majority of students were interested in radiology, though, unlike other studies, the numbers interested in the specialty significantly decreased on follow-up. The number considering radiology as a career showed an insignificant improvement post-module.

One could argue that the results are not unexpected given that radiological anatomy was the main focus of this teaching module and that clinical radiology was not the focus of the module. It is worth noting that formal assessment at the end of the imaging module was based on the taught content. However, the taught content was insufficient to complete all elements of the survey correctly. Notwithstanding, a reading list and links to online resources were given to the students to encourage further reading on the topic. By their own admission, however, these students do not have a high opinion of SDL. The absence of alteration in knowledge shows that a module in imaging anatomy as part of a year-long module in anatomy that included small group interactive sessions with clinical radiologists (which promoted SDL) did not appear to promote reading outside of the core curriculum. Overall, the findings in this study suggest that SDL techniques were not fully embraced in this cohort. Although most medical schools are emphasising vertical and horizontal integration and early introduction to clinical medicine, one could argue that integration of an imaging anatomy module into the anatomy curriculum is challenging in the pre-clinical context when students do not have experience of hospital procedures and basic awareness of the pivotal role of imaging in clinical diagnosis. There is an obvious potential for information overload when students are confronted with new concepts and terminology (angiography, cholangiography, contrast examinations, etc.) and the many minimally invasive and more invasive ways of acquiring these images (e.g. CT angiography, MR angiography, conventional angiography, MR cholangiopancreatography [CP] and endoscopic retrograde [ER] CP). Once clinical rotations commence, however, students gain first-hand experience of everyday medical practice and the roles of various medical specialties and subspecialties; learning is gradual through observation and tuition. These issues have implications for planning of vertical integration and curriculum design. Analysis of the study findings amongst anatomists and clinical radiologists who participated in the study led to the conclusion that education of medical undergraduates in imaging and clinical or applied anatomy is important, but that further modules in clinical and imaging anatomy should be integrated into modules in clinical medicine, surgery and radiology at a later stage in the undergraduate curriculum, when familiarity with imaging examinations will be much greater. Furthermore, these results suggest that students would benefit from dedicated radiation safety training. To this end, a focused module on radiation protection has been introduced into the final medical year and an assessment in radiation protection must be successfully completed prior to graduation.

A follow-up study of this cohort, to examine alterations in career aspirations, is planned to ascertain if student perceptions of radiology as a career change over time and to assess the impact, if any, that the 1st-year module had on these decisions.

In conclusion, the results of this study suggest that SDL is not favoured as a learning method for anatomy. Exposure of students to a module in imaging anatomy delivered by senior clinical radiologists improved basic knowledge regarding which procedures result in exposure to ionising radiation, but there was knowledge regarding relative amount of radiation exposure associated with individual modalities remained suboptimal post-module. In addition, knowledge of roles within a clinical radiology department showed little change on follow-up. A possible explanation is that students recall knowledge imparted in didactic lectures but do little reading around the subject.

## References

[1] Bergman EM, Verheijen IWH, Scherpbier AJJA, Van der Vleuten CPM, De Bruin ABH (2014). Influences on anatomical knowledge: The complete arguments. Clin Anat.

[2] Orsbon CP, Kaiser RS, Ross CF (2013). Physician opinions about an anatomy core curriculum: a case for medical imaging and vertical integration. Anat Sci Educ.

[3] Eisenstein A, Vaisman L, Johnston-Cox H, Gallan A, Shaffer K, Vaughan D, O’Hara C, Joseph L (2014). Integration of basic science and clinical medicine: the innovative approach of the cadaver biopsy project at the Boston University School of Medicine. Acad Med.

[4] Wijnen-Meijer M, ten Cate OTJ, van der Schaaf M, Borleffs JCC (2010). Vertical integration in medical school: effect on the transition to postgraduate training. Med Educ.

[5] Wijnen-Meijer M, ten Cate O, van der Schaaf M, Harendza S (2013). Graduates from vertically integrated curricula. Clin Teach.

[6] Alsaggaf S, Ali SS, Ayuob NN, Eldeek BS, El-Haggagy A (2010). A model of horizontal and vertical integration of teaching on the cadaveric heart. Ann Anat.

[7] Allen SS, Roberts K (2002). An integrated structure-function module for first year medical students: correlating anatomy, clinical medicine and radiology. Med Educ.

[8] Teichgräber UK, Meyer JM, Berens von Rautenfeld D (1996). Teaching applied anatomy to senior medical students with an emphasis on surgery and radiology. Surg Radiol Anat.

[9] Dettmer S, Tschernig T, Galanski M, Pabst R, Rieck B (2010) Teaching surgery, radiology and anatomy together: the mix enhances motivation and comprehension. Surg Radiol Anat 32(8):791–79 10.1007/s00276-010-0694-5 20623122

[10] Dettmer S, Schmiedl A, Meyer S, Giesemann A, Pabst R, Weidemann J, Wacker FK, Kirchhoff T (2013). Radiological anatomy—evaluation of integrative education in radiology. Röfo.

[11] McHanwell S, Davies D, Morris J, Parkin I, Whiten S, Atkinson M, Dyball R, Ockleford C, Standring S, Wilton J (2007). A core syllabus in anatomy for medical students—adding common sense to need to know. Eur J Anat.

[12] Sugand K, Abrahams P, Khurana A (2010). The anatomy of anatomy: a review for its modernization. Anat Sci Educ.

[13] Drake RL, McBride JM, Lachman N, Pawlina W (2009). Medical education in the anatomical sciences: the winds of change continue to blow. Anat Sci Educ.

[14] Collins JP (2008). Modern approaches to teaching and learning anatomy. BMJ.

[15] Chowdhury R, Wilson IDC, Oeppen RS (2008). The departments of radiology and anatomy: new symbiotic relations?. Clin Radiol.

[16] Burkill G, Francis I (2003). Trends in radiological anatomy teaching in the UK and Ireland. Clin Radiol.

[17] Jack A, Burbridge B (2012). The utilisation of radiology for the teaching of anatomy in Canadian medical schools. Can Assoc Radiol J.

[18] Ganske I, Su T, Loukas M, Shaffer K (2006). Teaching methods in anatomy courses in North American medical schools the role of radiology. Acad Radiol.

[19] Ahmed K, Rowland S, Patel VM, Ashrafian H, Davies DC, Darzi A, Athanasiou T, Paraskeva PA (2011). Specialist anatomy: Is the structure of teaching adequate?. Surgeon.

[20] Campbell IS, Fox CM (2012). Options for postgraduate anatomy education in Australia and New Zealand. N Z Med J.

[21] Elzubeir MA (2009). Graduate-entry medical students’ self-directed learning capabilities in a problem-based curriculum. Saudi Med J.

[22] Fanning DM, Chadwick G (2010). Preliminary analysis of demographics and learning attributes of graduate entry medical students. Ir J Med Sci.

[23] Miflin BM, Campbell CB, Price DA (1999). A lesson from the introduction of a problem-based, graduate entry course: the effects of different views of self-direction. Med Educ.

[24] Abraham RR, Fisher M, Kamath A, Izzati TA, Nabila S, Atikah NN (2011). Exploring first-year undergraduate medical students’ self-directed learning readiness to physiology. Adv Physiol Educ.

[25] Findlater GS, Kristmundsdottir F, Parson SH, Gillingwater TH (2012). Development of a supported self-directed learning approach for anatomy education. Anat Sci Educ.

[26] Harvey BJ, Rothman AI, Frecker RC (2003). Effect of an undergraduate medical curriculum on students’ self-directed learning. Acad Med.

[27] Lycke KH, Grøttum P, Strømsø HI (2006). Student learning strategies, mental models and learning outcomes in problem-based and traditional curricula in medicine. Med Teach.

[28] Premkumar K, Pahwa P, Banerjee A, Baptiste K, Bhatt H, Lim HJ (2013). Does medical training promote or deter self-directed learning? A longitudinal mixed-methods study. Acad Med.

[29] Gade S, Chari S (2013). Case-based learning in endocrine physiology: an approach toward self-directed learning and the development of soft skills in medical students. Adv Physiol Educ.

[30] Lee Y-M, Mann KV, Frank BW (2010). What drives students’ self-directed learning in a hybrid PBL curriculum. Adv Health Sci Educ Theory Pract.

[31] Bergman EM, Sieben JM, Smailbegovic I, de Bruin ABH, Scherpbier AJJA, van der Vleuten CPM (2013). Constructive, collaborative, contextual, and self-directed learning in surface anatomy education. Anat Sci Educ.

